# Learning the One-Electron Reduced Density Matrix at SCF Convergence Thresholds

**DOI:** 10.1021/acs.jctc.5c01564

**Published:** 2025-12-12

**Authors:** Bhaskar Rana, Nicolas Viot, Jessica A. Martinez B, Xuecheng Shao, Pablo Ramos, Michele Pavanello

**Affiliations:** † Department of Physics, 67206 Rutgers University, Newark, New Jersey 07102, United States; ‡ Department of Chemistry, 242613 Rutgers University, Newark, New Jersey 07102, United States; § Key Laboratory of Material Simulation Methods & Software of Ministry of Education, College of Physics, 12510 Jilin University, Changchun 130012, P. R. China; ∥ Division of Biology, Chemistry & Physics, Essex County College, Newark, New Jersey 07102, United States

## Abstract

Machine learning of the one-electron reduced density matrix (1-RDM) provides a computationally efficient surrogate to conventional electronic structure methods. In this work, we train models that map the electron−nuclear interaction potential to the 1-RDM with such an accuracy that predicted 1-RDMs deviate from fully converged ones by no more than a standard self-consistent field (SCF) threshold. Through targeted model optimization strategies, we demonstrate that training set sizes substantially smaller than those required in our previous work [ ShaoX.,  Nat. Commun. 14, 6281 (2023)]37805614 10.1038/s41467-023-41953-9PMC10560258 are sufficient to reach this accuracy. Furthermore, we introduce a force-correction algorithm that enables stable ab initio molecular dynamics powered by the machine learned 1-RDMs, extending the applicability of the surrogate electronic structure methods to molecules as large as biphenyl.

## Graphical abstract

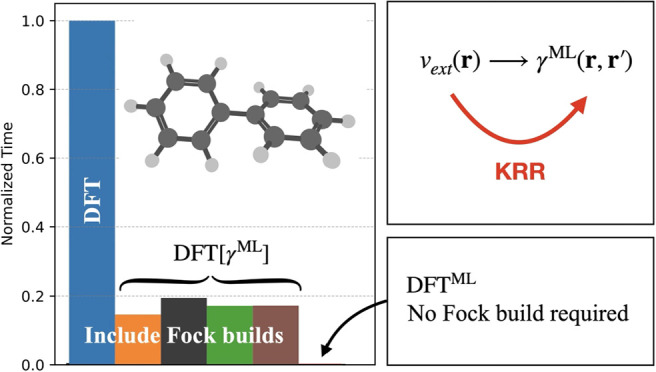

## 1 Introduction

Computational tools have become increasingly integral to the study and design of molecular and material’s properties. Yet, they often involve a significant computational cost, especially when applied to tackle the electronic structure of molecules and materials.^1,2^ As a result, there is a pressing need to develop methods that produce accurate electronic structures at reduced computational cost. Machine learning (ML) has emerged as a powerful way to generalize electronic structure methods and their outcome.^3–13^ These techniques have broadened the scope of quantum chemistry by enabling the study of systems larger in size compared to what is traditionally accessible while also extending simulation time scales, often without sacrificing chemical accuracy.^14,15^

The most useful ML models to date in chemistry and materials science have been those that target energies and atomic forces. That is, the ability of ML models to drive ab initio dynamics and other structural predictions based on a somewhat near-sighted awareness of atomic environments.^5,14–16^ However, recent efforts have begun to shift toward developing models that predict more fundamental quantities^6^ such as molecular wave functions,^17–22^ electron densities,^23–27^ effective Hamiltonians,^28,29^ reduced density matrices,^30–33^ Green’s functions^34^ and others.

Recently, several groups have advanced the idea of generating ML models that predict prototypical QM quantities with such accuracy that the use of the original QM method may become unnecessary.^20,23,30,35,36^ Along these lines, our group has demonstrated the feasibility of learning the map between the electron−nuclear attraction (external) potential and the one-electron reduced density matrix (1-RDM).^30^ This fundamental map implies that the 1-RDM, γ̂, is a functional of the external potential, v̂. Consequently, the functional γ̂[v̂] can be learned by regression models due to the inherent single-valuedness of the functional dependence.

Learning the 1-RDM is an interesting alternative to learning other quantities, such as the electron density or the electronic wave function. The 1-RDM provides direct access to any 1-electron operator, including nonmultiplicative ones like the kinetic energy. Thus, the information directly accessible from the 1-RDM is much wider than that accessible from the density alone. The 1-RDM is of much reduced size compared to the wave function. It merely scales quadratically with system size (it is a matrix). Additionally, the 1-RDM has well-known and easily imposed *N*-representability conditions. Specifically, the 1-RDM’s eigenvalues should be between zero and one (or two, for spin-compensated systems) and sum up to the number of electrons.

In ref 30., we showed that it is indeed possible to build models for the 1-RDM such that the predicted 1-RDMs are very accurate. The accuracy reached is in the order of typical SCF threshold values (∼10^−6^ Ha). The workhorse to reach such an accuracy in learning the functional relation γ̂[v̂] is the kernel ridge regression (KRR). The resulting models are somewhat narrow in scope, as they are molecule specific, method specific and computational setting specific (e.g., temperature, basis set). For example, a model can be developed for the water molecule at 300 K described by the Kohn−Sham DFT method employing the B3LYP functional^37^ using the 6−31G* basis set. Models for an array of DFT methods and wave function-based methods were also developed.

Already at conception, the method presented some computational barriers: (1) The training set size was found to scale cubically with system size. This put a severe bound on the size of the molecules that could be tackled in ref 30. (2) A multi stage learning protocol was required to achieve accurate 1-RDMs (specifically, to be clarified later, γ-learning followed by δ-learning). Both limitations meant that only midsized molecules could be tackled (such as benzene and propanol).

The goal of this work is to deliver accurate and predictive surrogate electronic structure methods based on machine learning models. We do so by critically assessing the models developed in ref 30., comparing them with a range of available alternatives, and optimizing both the workflow and the associated hyperparameters. We identify improved models that enhance the accuracy and computational efficiency of the previously developed framework. Specifically, for the task of learning the v̂ → γ̂ map and extracting molecular properties from it, we aim at answering the following questions: (1) Are there regression methods that match or surpass the accuracy of KRR? (2) Can accurate models be achieved with smaller training sets? (3) Can these ML methods be employed to perform stable ab initio molecular dynamics (AIMD) simulations for systems of larger size than considered before? In the sections that follow, we address each of these questions systematically. In particular, we show that for AIMD, the computation of atomic forces requires a nuanced approach.

This work focuses on mean-field approaches, in particular DFT, which remains the primary target of most ML models, including ML potentials. Nonetheless, as shown in our previous work, the framework and analysis developed here are expected to be largely transferable to learning 1-RDMs from correlated wave function methods.

## 2 Methods

The key component of the workflow is the 1-RDM, denoted as γ̂. It is a fundamental quantity in quantum chemistry as it encapsulates essential electronic structure information. Given an *N*-electron wave function, Ψ, γ̂ is formally defined as (disregarding spin for the moment):

1 $$\gamma \left(r,r^{\prime }\right)=N\int \Psi^{*}\left(r^{\prime },r_{2},...,r_{N}\right)\Psi \left(r,r_{2},...,r_{N}\right)dr_{2}...dr_{N}$$

In mean field methods, where the wave function is restricted to be a single Slater Determinant, the 1-RDM takes a spectral form, γ­(**r**, **r**′) = ∑_*i*_ *n*_*i*_ ϕ_*i*_(**r**)­ϕ_*i*_(**r**′). For DFT methods, and specifically Kohn−Sham DFT, the available 1-RDM is usually the Kohn−Sham 1-RDM, often denoted as γ̂_*s*_. While γ̂_*s*_ is an important quantity, it is not strictly equal to the true, interacting 1-RDM. In what follows, we will set up learning strategies for the 1-RDM of DFT methods, that is to be precise, γ̂_*s*_.

The 1-RDM is then related to the external potential (v̂), which is usually considered to be space-local. However, in practice it could be nonlocal for example when pseudopotentials are employed.^39^ In this work, we consider all the electrons in the system (core and valence), and thus we employ the full Coulomb potential defined as

2 $$v\left(r\right)=-\sum_{I=1}^{\#\mathrm{of}\;\mathrm{atoms}}\frac{Z_{I}}{\left|r-R_{I}\right|}$$

where {*Z*_*I*_} and {**R**_*I*_} are the nuclear charges and positions.

The theorems of reduced density matrix functional theory^40^ allow us to rigorously map the external potential with the 1-RDM. The main idea is that one can rigorously consider the 1-RDM to be a functional of the external potential, γ̂[v̂], allowing us to “machine learn” (ML) the functional dependence of these quantities. Two key maps are

3 mapγ:v̂→γ̂

4 mapδ:γ̂→E,F,⟨Ô⟩

Here, *E* and *F* denote the electronic energy and atomic forces, respectively. ⟨*Ô*⟩ represents the expectation value of any operator.

The main goal of this work is to learn the above maps. Hereafter, we refer to ML models for map γ as “*γ*-learning,” and for map δ as “δ-learning.” The additional nomenclature γ + δ reflects the practical fact that map δ is typically learned from the predicted 1-RDMs produced by γ-learning (see workflow in Figure 1). In this framework, the ML models replace the computationally demanding self-consistent field (SCF) procedure required by mean-field methods such as Kohn−Sham DFT and Hartree−Fock. Moreover, if the learned 1-RDM is derived from wave function-based methods, the steeply scaling post-Hartree−Fock methods would also be effectively “learned away.”

**Figure 1:**
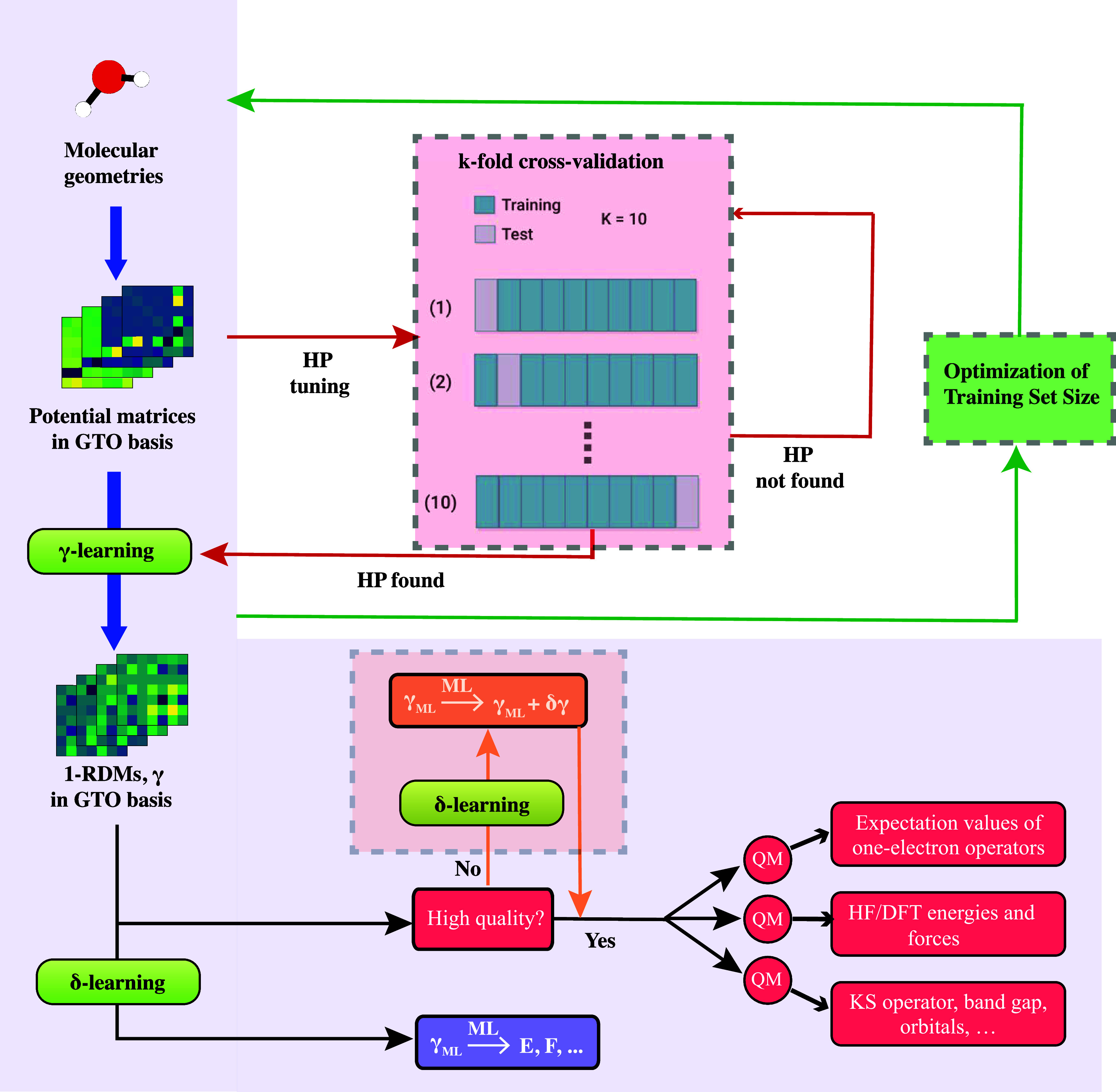
QMLearn workflow^38^ developed in this work. The 1-RDM of molecules is learned in the GTO basis as a functional of the external, electron−nuclear potential which is also expressed in terms of GTOs. The models implements two relations (maps): γ-learning targets 1-RDMs as a functional of the external potential. δ-learning targets energies, forces and other properties, as functionals of the 1-RDM. Additionally, δ-learning can also be used for purely computational purposes, i.e., to learn the error between the predicted 1-RDM from γ-learning and the target 1-RDM. See text for further details. Modifications to the original workflow are highlighted in the panels and include an iterative framework using k-fold cross-validation to fine-tune hyperparameters (α and Γ denoted in the figure as HP) and reduction of the training data set size.

A key advantage of learning the 1-RDMs of mean-field methods is that, in addition to one-electron operators, both energy and forces are pure functionals of the 1-RDM. Thus, with sufficiently accurate predictions of the 1-RDM, one can directly evaluate energy and atomic forces from the predicted 1-RDM. In contrast, wave function-based methods do not provide energy functionals that depend solely on the 1-RDM. Although the existence of a functional relationship *E*[γ̂] is formally established, its explicit dependence is unknown in the general case. This limitation is circumvented by introducing a second learning step (via the previously defined map δ) which enables the evaluation of energies and forces despite the absence of an explicit functional form.

γ- and δ-learning are achieved with kernel ridge regression (KRR), where the external potential serves as the input and the 1-RDM as target. Namely,

5 $$\mathrm{\gamma ̂}\left[\mathrm{v̂}\right]=\sum_{i=1}^{N_{\mathrm{sample}}}{\mathrm{\beta ̂}}_{i}K\left({\mathrm{v̂}}_{i},\mathrm{v̂}\right)$$

where {v̂_*i*_} represent training set of external potentials. The kernel matrix, $K_{\mathrm{ij}}=K\left({\mathrm{v̂}}_{i},\hat{v_{j}}\right)$ is the kernel measure between two potentials (feature vectors). {β̂_*i*_} are KRR weights obtained through inversion of the regularized kernel matrix. We note that β̂_*i*_ is a quantity of the same shape as γ̂.

An interesting application of δ-learning is for purely computational gain. Specifically, it can be used to refine the results of γ-learning by taking the γ predicted in the γ-learning stage as input features and the original target γ̂ as new target (see orange inset above “High quality?” in the workflow, Figure 1). In both our previous and current work, we have found that this approach improves prediction accuracy. We denote by γ + δγ the refined prediction obtained from γ-learning followed by δ-learning.

Three models summarize our approach where QM indicates any electronic structure method (typically, if a DFT method is targeted, QM will be replaced by the acronym corresponding to the exchange-correlation functional approximation):

1. **QM** ^**ML**^[γ^*p*^]: Predicts properties and energies directly using 1-RDMs predicted from γ-learning, γ^*p*^.
2. **QM** ^**ML**^[γ^*p*^ + δγ^*p*^]: Refines predictions by correcting 1-RDMs with δ-learning, γ^*p*^ + δγ^*p*^.
3. **QM** ^**ML**^: Predicts the 1-RDM using γ-learning and then δ-learning (i.e., learning map δ in eq 4) for energies and forces.

To distinguish the target 1-RDM from the predicted one, we indicate from now on the predicted with a “p” superscript.

These methods establish robust and efficient surrogates to traditional quantum mechanical methods, reducing computational costs without compromising accuracy or breath of scope. As for every ML model, key to the success of these methods is the generation of the training set and the use of optimal hyperparameters. In the next section, we discuss our strategy for hyperparameter tuning and also touch upon the possibility to use an optimal training set of reduced size compared to the results presented in our previous work.^30^

## 3 Protocol Optimization Strategy

### 3.1 Machine Learning Models

The goal is to learn the γ and δ maps presented before. To do so, we considered several regression models using their default hyperparameters. Their benchmark against the target ab initio method (to keep the computational cost low, we chose LDA functional for this test) is presented in Tables S1−S3. We found that, among the models tested, kernel ridge regression (KRR) and orthogonal matching pursuit (OMP)^41^ perform well for the γ-learning step, while linear regression and Gaussian process regression (GPR)^42^ proved most effective for the δ-learning step. However, while GPR and OMP performed well with smaller molecules (e.g., water, carbon dioxide, and ammonia), they exhibited substantial errors when applied to learning the 1-RDM of larger molecules likely because training these models require an optimization process associated with a substantial computational cost and dependence on initial conditions (e.g., for GPR, the input kernel). In contrast, the combination of KRR for the γ-learning step and linear regression for the δ-learning step consistently delivered the most reliable performance across all molecules tested and the computationally cheapest training. Following these comparisons, KRR for γ learning and linear regression for δ learning were ultimately identified as the most promising models and are the target of subsequent hyperparameter tuning efforts.

We stress that in the simulations run for selecting the models, we did not optimize their hyperparameters, default parameters were employed. For water, we carried out an analysis of the performance of the methods when the regularization hyperparameter (e.g., the α of KRR and similar for other models) is chosen appropriately, see Table S2. The analysis confirms the trends discussed above and also shows that certain models, in particular the available ones based on deep learning (MLP in Table S2), exhibited convergence issues, regardless of depth, activation function, or propagation function. This is possibly related to the comparatively small sample sizes we require for our regressions, particularly for H_2_O and CO_2_ had only 27 samples. Models that performed very poorly (e.g., that demonstrated some or all of the issues discussed) were not further considered and are not included in the tables. These are stochastic gradient descent,^19,43^ Bayesian ARD,^44^ and logistic regression,^45^ among others; gradient boosting ensemble methods;^46^ and multioutput techniques.^47^ Thus, these were not considered moving forward.

### 3.2 Hyperparameter Tuning

Once the models are selected, the next step is to optimize different kernel types and their hyperparameters with the goal of further improving the models and reducing RMSE values. This was achieved by performing *k*-fold cross-validation via both grid and randomized searches over the hyperparameters (see light pink inset in Figure 1). The entire data set was used and subdivided into 5 “folds” for α, representing the *L*2-regularization for the kernel, and Γ, which acts as the scaling factor for several of the KRR kernels (vide infra). The resulting workflow is visualized in Figure 1. The scoring metric employed was the negative root-mean-square error, as it proved to be the most stable and consistent across all molecules tested during repeated testing.

Two different procedures were used for hyperparameter optimization of the kernel ridge regressor, depending on the size of the molecules. For smaller molecules, the parameter space included the kernel type and α as simultaneous targets. We explored several kernels, including the linear (LIN), radial basis function (RBF), polynomial (POL), Laplacian, and sigmoid kernels.

The mathematical definitions of these kernels are as follows: *K*_LIN_(**x**_*i*_, **x**_*j*_) = **x**_*i*_^τ^ **x**_*j*_, *K*_RBF_(**x**_*i*_, **x**_*j*_) = exp­(−Γ∥**x**_*i*_ − **x**_*j*_∥^2^), and *K*_POL_(**x**_*i*_, **x**_*j*_) = (Γ**x**_*i*_^τ^ **x**_*j*_ + *c*)^*d*^. Where **x**_*i*_^τ^ **x**_*j*_ is the dot product of the input vectors, *c* is a constant (typically *c* ≥ 0), and *d* is the degree of the polynomial. In our analysis, we employed the default parameter values of *d* = 3 and *c* = 0, without applying any hyperparameter optimization for these. To clarify their use, in our workflow these kernels are applied to distinct feature representations in the two learning steps. For γ-learning, the feature vectors **x**_*i*_ and **x**_*j*_ correspond to the external potential v̂, while for δ-learning, they correspond to γ itself.

In our tests, the RBF kernel was consistently selected by both grid and randomized searches. For larger molecules, due to the relatively larger size of the training set, all kernels were optimized independently, instead of being included in the parameter space. We found that, other than for the linear kernel, all other kernels seemed to favor values of α close or equal to zero. The Γ parameter was initially left at its default value, which is $\frac{1}{N_{\mathrm{features}}}$ , where *N*_features_ is the total number of features in the data set. To ensure the best results, we also optimized Γ using cross-validation. Interestingly, the optimization process repeatedly converged to values nearly identical to the default, $\frac{1}{N_{\mathrm{features}}}$ , confirming that the default setting is already effective. As a result, further optimization of Γ was unnecessary.

From these initial attempts, the top three performing kernels were retained: the linear (LIN), radial basis function (RBF), and polynomial (POL) kernels.

The outcome of these testing was to retain the linear, polynomial, and RBF kernels, all with α values of zero and with Γ values as discussed above. These formed the basis of subsequent stability and extrapolation testing, and comparison to the previously published model.^30^

### 3.3 Training Set Generation

The strategy for generating training data sets aims to minimize computational overhead while still providing an exhaustive sampling. Generating training sets is in fact the most computationally intensive task of ML and data-driven models for quantum chemistry and material science.^48–50^ Our approach is to completely avoid AIMD simulations in this step because these inherently produce many redundant training geometries which would need to be sifted by clustering methods. Instead, we perform a normal-mode analysis at the equilibrium geometry for each molecule. Normal mode displacements are then sampled with a normal distribution along the vibrational modes (there are *N*_vib_ vibrational modes) of variance proportional to *k*_B_ *T* where *T* is a target temperature for the ML models. That is, the ML models will perform best if used in a regime consistent with the target *T*. In this work, we set *T* = 300 K for all cases considered.

The computational complexity resides almost entirely in the generation of the training set rather than in the training of the KRR models. This is because kernel methods require, in most instances, merely the solution of a linear problem of leading dimension the size of the training set. To find an appropriate number of training structures, we simply grow the training set, train the model and test until a satisfactory comparison against the target electronic structure method is achieved. In our past work^30^ we generally required $O\left(N_{\mathrm{vib}}^{3}\right)$ training points. We will see later that although an $O\left(N_{\mathrm{vib}}^{3}\right)$ training set size is required for small molecules, larger molecules require a training set of much reduced size.

### 3.4 Purification of Predicted 1-RDMs

The 1-RDM predicted by our machine learning model may not strictly satisfy the N-representability conditions. In its spectral representations, the 1-RDM can be written as

γ^=∑ini|ϕi⟩⟨ϕi| 6

where *n*_*i*_ are the natural orbital occupation numbers and |ϕ_*i*_⟩ are the corresponding natural orbitals. We impose aufbau occupations by performing a single diagonalization of the predicted 1-RDM. For the molecular systems considered in this work, a single diagonalization does not adversely affect computational scaling. Should larger systems be considered, iterative procedures like McWeeny’s purification can be implemented.

### 3.5 Atomic Forces

Atomic forces in electronic structure methods typically exploit the Hellmann−Feynman theorem. The theorem states, in its generalized sense,^51,52^ that if the energy functional, *E*, has been variationally optimized with respect to the quantity of reference (the Kohn−Sham orbitals for Kohn−Sham DFT, the density, ρ­(**r**) = γ­(**r**, **r**), for orbital-free DFT, the 1-RDM, γ̂, for 1-RDMFT) then, in the absence of the so-called Pulay terms,^53^ the atomic forces are simply given by **F**_*I*_ = ∫ρ­(**r**)**∇**_*I*_ *v*({**R**_*I*_}; **r**), where **∇**_*I*_ is the gradient wrt the position of nucleus *I*, **R**_*I*_, and *v*({**R**_*I*_}; **r**) is defined in eq 2.

What happens when the energy functional is not variationally optimized? Formally, for 1-RDMFT, the forces are

$$F_{I}=\int \rho \left(r\right)\nabla_{I}v\left(\left\{R_{I}\right\};r\right)dr+Tr[\frac{\delta L}{\delta \mathrm{\gamma ̂}}\nabla_{I}\mathrm{\gamma ̂}]$$ 7

For mean field methods, which make use of orthogonal orbitals, {ϕ_*i*_(**r**)}, the Lagrangian takes the form (disregarding spin for now),

8 $$L=E\left[\mathrm{\gamma ̂}\right]-\sum_{i}\lambda_{i}\left(\left\langle \phi_{i}\right|\phi_{i}\rangle -1\right)-\mu \left(\sum_{i}n_{i}-N\right)$$

with the Lagrange multipliers defined as^54,55^ λ_*i*_ = *ε*_*i*_ *n*_*i*_, *n*_*i*_ being the 1-RDM occupation numbers and *ε*_*i*_ the orbital energies.

Knowing that the Fock operator is defined as $\mathrm{f̂}=\frac{\delta E}{\delta \mathrm{\gamma ̂}}$ , and assuming aufbau occupations (*n*_*i*_ = 1 for *i* spanning occupied orbitals and *n*_*i*_ = 0 for virtuals) we find $\delta L=\sum_{i}\frac{\delta L}{\delta \phi_{i}}\delta \phi_{i}$ , which results in the following force

$$F_{I}=\int \rho \left(r\right)\nabla_{I}v\left(\left\{R_{I}\right\};r\right)dr+Tr\left[\mathrm{f̂}\nabla_{I}\mathrm{\gamma ̂}\right]-\underset{i}{\sum }\lambda_{i}\left(\left\langle \phi_{i}\right|\nabla_{I}\phi_{i}\rangle +\left\langle \nabla_{I}\phi_{i}\right|\phi_{i}\rangle \right)$$ 9

The last term on the rhs is related to the derivative of the energy-weighted density matrix ρ̂_ε_ = ∑_*i*_ε_*i*_ *n*_*i*_ |ϕ_*i*_⟩⟨ϕ_*i*_| which can be derived from the Fock operator and the 1-RDM as ρ̂_ε_ = *f̂*γ̂. Therefore, the above equation can be recast as follows,

$$F_{I}=\int \rho \left(r\right)\nabla_{I}v\left(\left\{R_{I}\right\};r\right)dr+Tr\left[\left(\mathrm{f̂}-{\mathrm{f̂}}^{\prime }\right)\nabla_{I}\mathrm{\gamma ̂}\right]$$ 10

where *f̂*′ is a Fock operator whose spectral decomposition yields the orbitals {ϕ_*i*_}. Crucially, due to the assumed nonvariational nature of the 1-RDM (as it is the outcome of a ML model), the {ϕ_*i*_} orbitals do not originate from *f̂*. *f̂*′ can be interpreted in terms of an inverse problem: having a set of orbitals which we impose to be noninteracting v̂′-representable, we can find a Fock operator *f̂*′ = −1/2 ∇^2^ + v̂′ whose eigenfunctions are the same orbitals.

The core task is then to find δ*f̂* = *f̂* − *f̂*′ which can be done in a number of ways. One is via linear-response theory, finding $\delta \mathrm{f̂}=\frac{\delta \mathrm{f̂}}{\delta \mathrm{\gamma ̂}}\delta \mathrm{\gamma ̂}=\mathrm{K̂}\delta \mathrm{\gamma ̂}$ with the kernel matrix, *K*_μνστ_ = ⟨μν|*r*_12_^−1^ + *f*_*xc*_|στ⟩. Alternatively, we found one can simply use the diagonalized Fock operator built from γ̂. Namely, *f̂*′ = diag­(*f̂*[γ̂]). While this adds a diagonalization step to the algorithm, a single diagonalization, rather than a full SCF still results in a low-prefactor, computationally cheap method.

## 4 Computational Details

**Table 1:**
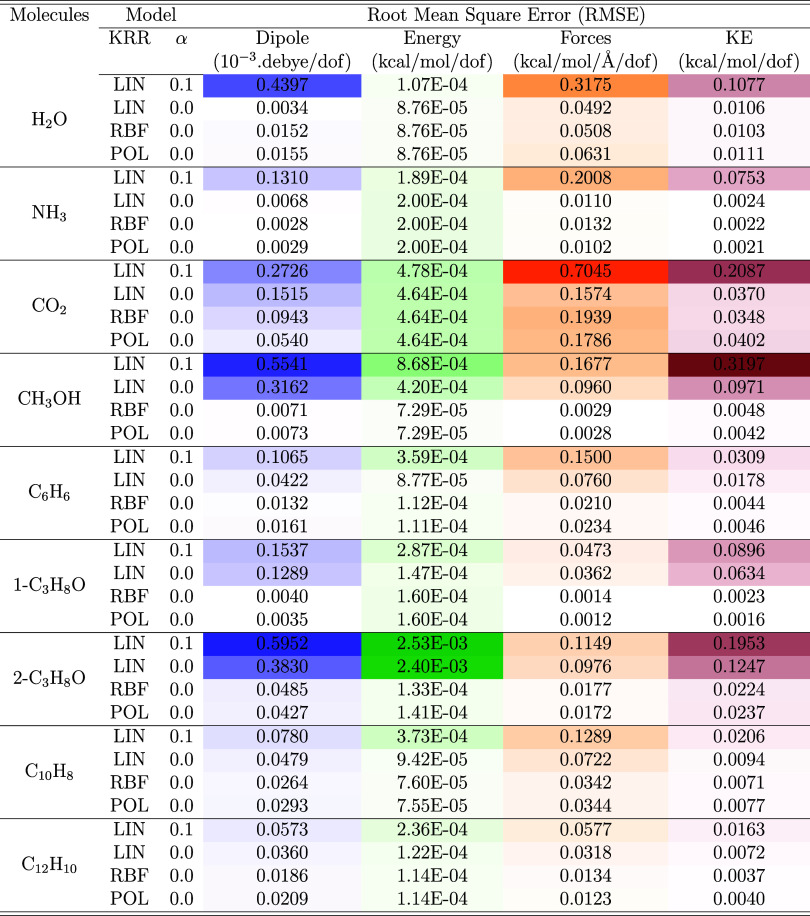
Root Mean Square Error (RMSE) for Dipole Moment, Energy, Forces, and Non-Interacting Kinetic Energy (KE) Predicted by the γ-Learning Step with Several KRR Kernels (LIN: Linear, RBF, and POL: Polynomial) with Respect to Target B3LYP Results for All Molecules^a^

a α is the kernel regularization parameter. All models use the same training set size. Color coding intensity is related to the magnitude of the RMSE per column. LDA results are shown in Table S4.

**Table 2:**
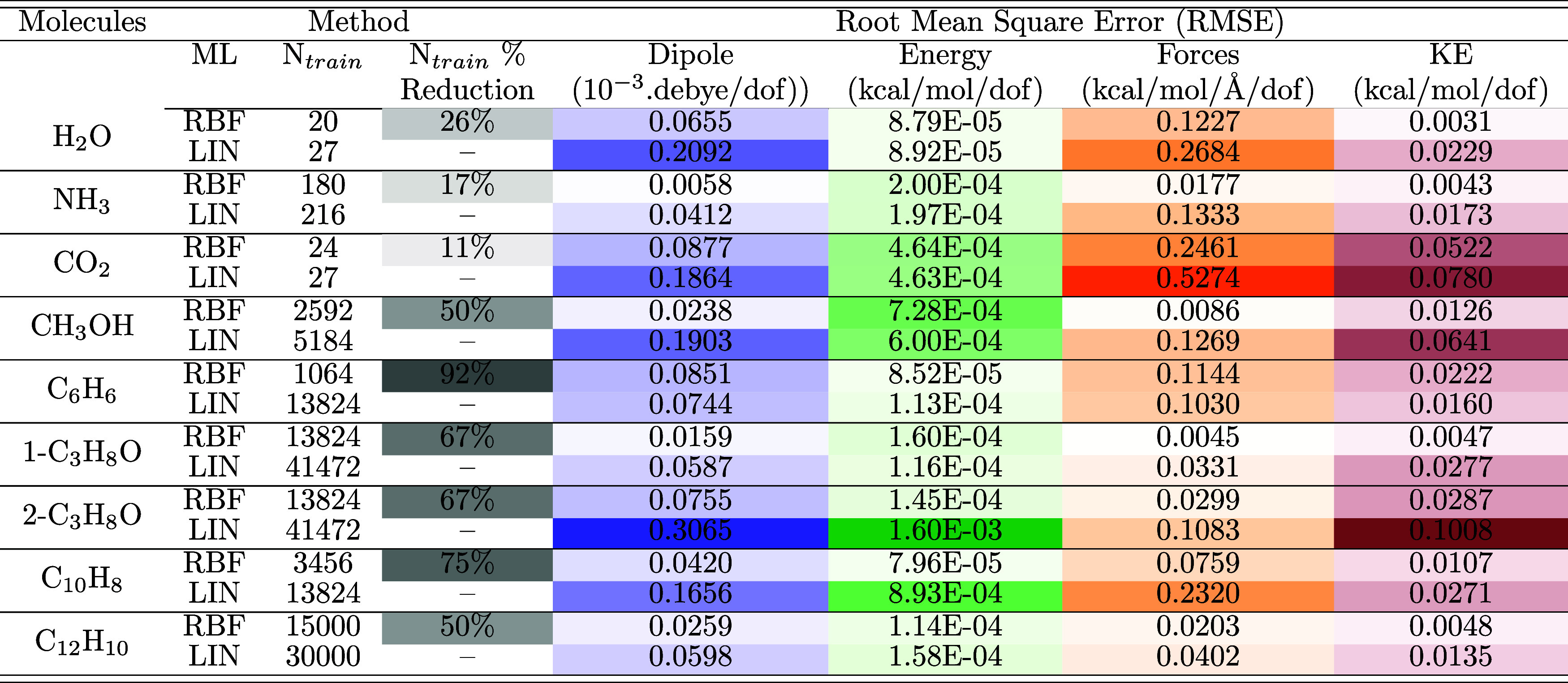
Same Quantities as Table 1 and Additionally the Training Set Size Used^a^

a The RBF model used a reduced training set size compared to Table 1. Results from two ML models are compared: B3LYP^ML^[γ_RBF,α = 0.0_^*p*^] (RBF) and B3LYP^ML^[γ_LIN,α = 0.1_^*p*^ + δγ_LIN_^*p*^] (LIN). LDA results are shown in Table S5.

The specific models described above were tested on a diverse set of molecules, varying in complexity, rotational degrees of freedom, and size: H_2_O, NH_3_, CO_2_, CH_3_OH, C_6_H_6_, 1-C_3_H_8_O (1-propanol), 2-C_3_H_8_O (2-propanol), C_10_H_8_ (naphthalene), and C_12_H_10_ (biphenyl).

Whenever possible, the training sets were borrowed from a database developed by Shao et al.^30^ For the newly studied molecules, naphthalene (C_10_H_8_) and biphenyl (C_12_H_10_), we generated the training sets using exactly the same protocol. PySCF^56^ was used to compute all the necessary quantities including v̂ and γ̂ in the Gaussian type orbital (GTO) basis. The cc-pVTZ basis set was employed for water, ammonia, carbon dioxide. The 6−31G* basis set was employed for all other molecules. The models were trained on Kohn−Sham DFT with the LDA and B3LYP exchange-correlation functionals.^37,57^

The test data sets were generated via AIMD simulations performed with the same DFT levels of theory using PySCF and driven by Atomic Simulation Environment (ASE) toolkit.^58^ Each simulation employed the velocity Verlet integrator with a 0.5 fs time step under microcanonical (NVE) conditions, and initial momenta were assigned according to a Maxwell−Boltzmann distribution at a target temperature of 300 K for an overall duration of 2 ps. From the last 1 ps of dynamics, 100 geometries were selected at equally spaced time intervals.

Regressions employed models from Sci-Kit Learn, and cross-validation was performed following established protocols (k-fold). Further details about the algorithm used can be retrieved from the QMLearn code used in this study, publicly available on GitHub.^38^

## 5 Results and Discussion

The protocol optimization strategy was implemented and tested on Kohn−Sham DFT using the LDA and B3LYP exchange-correlation functionals.^37,57^ Once the optimized ML model is selected, we present machine learned hybrid functional (B3LYP^37^) models for an array of molecules. We conclude this section with an analysis of the computational costs involved in comparison to the target DFT method.

### 5.1 KRR Kernels and Hyperparameters for γ-Learning

We focus our analysis on the performance of KRR for the γ-learning step. Table 1 summarizes the root-mean-square error (RMSE) for KRR with different kernels on the test data set (see Section 4), comparing various molecular properties including dipole moment, energy, forces, and noninteracting kinetic energy (KE).

The linear kernel (LIN) with regularization parameter α = 0.1 (used in the models of ref 30.), consistently predicts lower quality RMSEs compared to the other kernels considered (POL and RBF) and compared to using α = 0.0. We note that the value α = 0.0 consistently showed improved performance across all considered molecules. We thus posit that that the optimal value of α is system independent.

Our analysis uncovers three important conclusions about the RBF kernel employed in the γ-learning step:

- it is superior to the LIN kernel,
- at the γ-learning step, it produces a comparable accuracy to the LIN kernel when the latter is augmented by the δ-learning step,
- it requires a significantly reduced training set size compared to the LIN kernel.

Table 2 presents a comparison between the optimized model, which relies exclusively on γ-learning (B3LYP^ML^[γ_RBF,α = 0.0_^*p*^]), and the model originally presented in ref 30. that uses γ + δ-learning (B3LYP^ML^[γ_LIN,α = 0.1_^*p*^ + δγ_LIN_^*p*^]). Clearly, the optimized model, B3LYP^ML^[γ_RBF,α = 0.0_^*p*^], delivers results on-par with the previous model for all molecules considered. A crucial observation is that while the original model required an additional, refining δ-learning for the 1-RDM, the new, optimized model delivers as accurate results with a single KRR regression (i.e., in our nomenclature, γ-learning). This not only reduces the computational cost of the evaluation of the model but also gives us an opportunity to further optimize the size of the training set. Similar analysis with LDA functionals are presented in Table S4.

### 5.2 Training Set Optimization

With KRR using the linear kernel, the size of the training set required to achieve SCF-threshold accuracy was empirically found to scale like $O\left(N_{\mathrm{vib}}^{3}\right)$ , where *N*_vib_ denotes the number of vibrational modes.^30^ In addition to the RMSEs, Table 2 presents the training set size and its percent reduction when B3LYP^ML^[γ_RBF,α = 0.0_^*p*^] is employed. The results clearly show that the optimized model with the RBF kernel maintains or improves accuracy while requiring significantly fewer training points.

Figure 2 shows more clearly that in fact the training set size scaling should decrease with system size. In the limit of a rigid molecule, due to the constrained geometry, we expect the training set to grow linearly with *N*_vib_ (or equivalently the number of atoms). When the molecules display conformational freedom, we expect the training set to asymptotically grow like *N*_vib_ × *N*_conf_ where *N*_conf_ is the number of relevant molecular conformations. This is clearly seen in Figure 2 where for methanol a training set of size exactly *N*_vib_ × 3 is needed. Moving to naphthalene, the training set size is already considerably smaller than *N*_vib_^3^.

**Figure 2:**
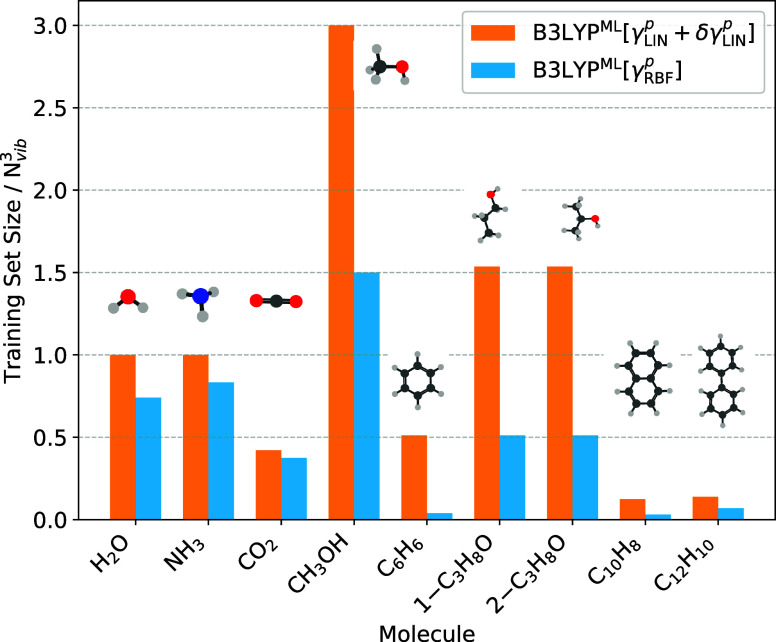
Normalized training data set sizes for all molecules investigated in this work. All values are normalized by *N*_vib_^3^, where *N*_*vib*_ is the number of vibrational modes. For raw values, see Table 2. The LDA results are shown in Figure S7 and Table S5.

The accuracy of the predicted electronic energy as a function of the employed training set size are reported in Figure 3 for three ML models: B3LYP^ML^[γ_LIN_^*p*^], B3LYP^ML^[γ_LIN_^*p*^ + δγ_LIN_^*p*^], and B3LYP^ML^[γ_RBF_^*p*^] and three molecules: benzene, methanol and 2-propanol. Across all the molecules, these ML models exhibit similar performance, achieving chemical accuracy rapidly. Benzene reaches this accuracy with just 50 structures, while methanol and 2-propanol require around 100 structures. Among the three models, the optimized B3LYP^ML^[γ_RBF_^*p*^] consistently outperforms the others in achieving SCF-threshold accuracy. Instead the B3LYP^ML^[γ_LIN_^*p*^] model falls short, even with training sizes as large as 10,000 structures. The B3LYP^ML^[γ_LIN_^*p*^ + δγ_LIN_^*p*^] model performs better than the B3LYP^ML^[γ_LIN_^*p*^] model but it is generally less accurate (and more expensive) than the B3LYP^ML^[γ_RBF_^*p*^] model. Similar qualitative results are obtained for LDA functionals (see Figure S8).

**Figure 3:**
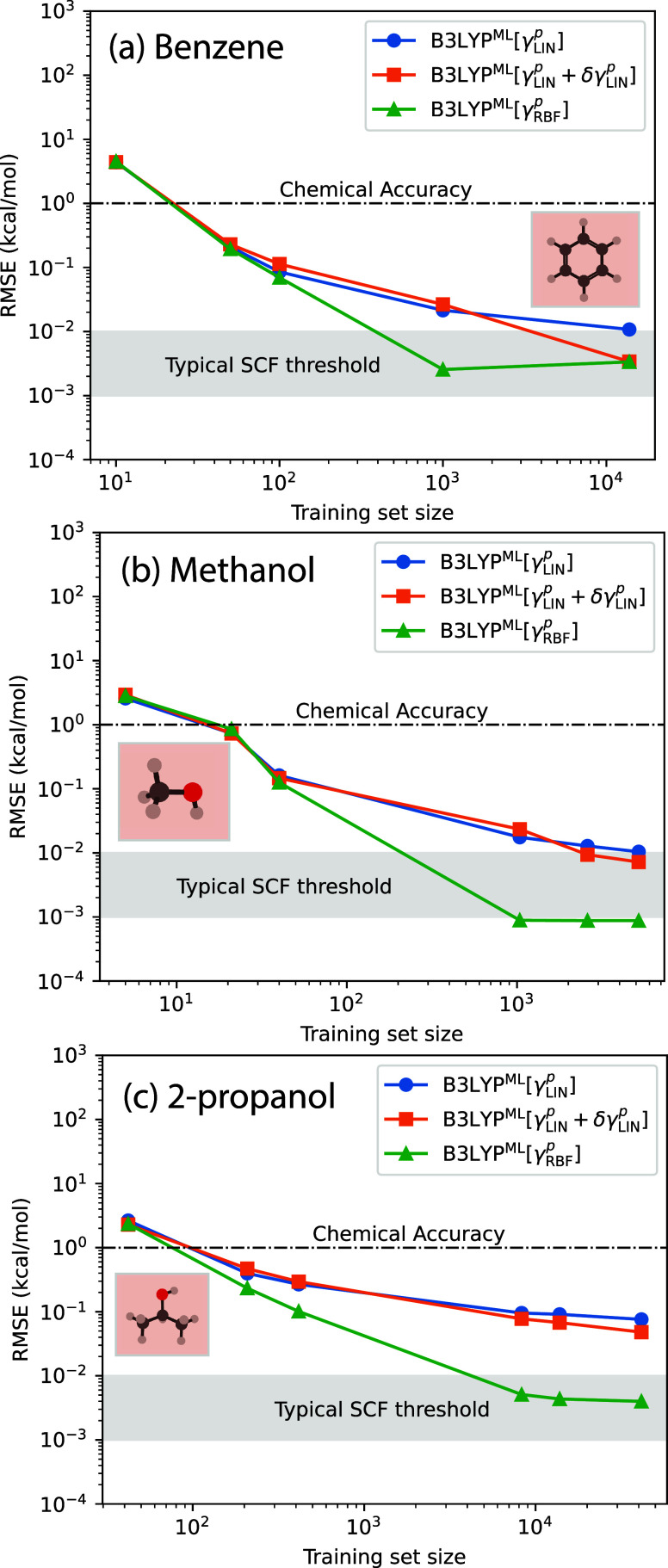
Energy RMSE for various ML models as a function of training set size for (a) benzene (C_6_H_6_), (b) methanol (CH_3_OH), and (c) 2-propanol (2-C_3_H_8_O). The gray shaded area represents SCF-threshold accuracy, i.e., the accuracy we require from a surrogate model. LDA results are shown in Figure S8.

A training set consisting of tens of thousands of structures for a single molecule may seem impractical. However, two points are worth noting: (1) such data set sizes are not computationally expensive, because KRR prediction scales linearly with the number of training structures, and (2) accurate fits of molecular energy surfaces for spectroscopic applications which typically require tens to hundreds of thousands of geometries.^59^

It is natural to wonder what are the limits of our approach. Can the method, as is, be applied to macromolecules or condensed phases? From a computational standpoint the answer is “probably yes” simply because of the expected linear relationship between the system size and the training set size. However, the costs associated with generating a training set for a macromolecule may be completely impractical. Thus, it is more likely that the method presented here will need to be modified in some ways (folding in techniques such as quantum embedding) to allow it to approach large systems and condensed phases. Work along these lines is ongoing in our lab.

### 5.3 Extrapolative Behavior of the ML Models

We now assess the reliability of these optimized models across configuration spaces, both within and beyond the subspace spanned by the training set, by evaluating their ability to predict the HOMO−LUMO gap for water (Figure 4a) and biphenyl (Figure 4b). To achieve this, we systematically varied the displacement along a relevant internal coordinate (the OH stretching coordinate for water and the dihedral for biphenyl), generating geometries that span and extend beyond the original training space. The results show that for both water and biphenyl, the HOMO−LUMO gap can be predicted to a reasonable accuracy, closely recovering the trends, even far away from the configuration space spanned by the training set. This conclusion was also reached in our previous work, and here is confirmed also for the optimized models. In fact, we see that B3LYP^ML^[γ_RBF_^*p*^] systematically improves upon the performance of B3LYP^ML^[γ_LIN_^*p*^ + δγ_LIN_^*p*^]. We obtain similar results when the models are trained with the LDA functional (see Figure S1).

**Figure 4:**
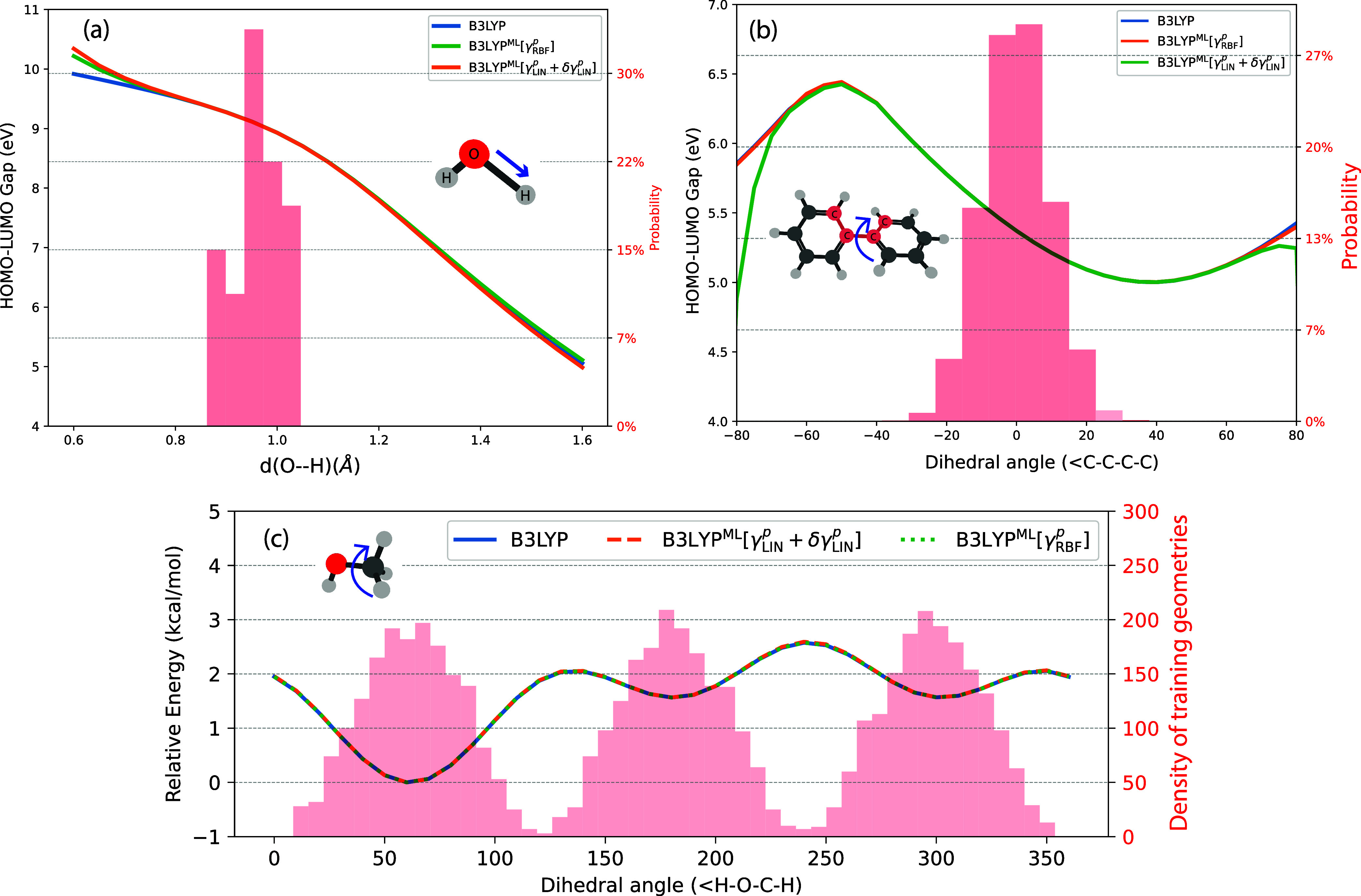
HOMO−LUMO gap for (a) a water molecule with geometries along the O−H bond stretch and (b) a biphenyl (C_12_H_10_) molecule with geometries following the dihedral angle (<C−C−C-C) twist, measured relative to the equilibrium structure (which corresponds to a dihedral angle of 38.49°). (c) Conformational energy barrier for a methanol molecule for a rotation around the C−O bond. Results from two ML models are compared with the B3LYP calculations. The histograms indicate the configuration space spanned by the training set structures (with y-scales indicated by "probability" for panels a and b and "density of training geometries" for panel c).

It is interesting to cast the results in terms of the ability of the ML models to interpolate and extrapolate. Clearly, the models considered can interpolate quite well. They can also extrapolate fairly well with a deviation that grows in proportion to the distance to the configuration space spanned by the training set. We should clarify that a possible reason for such an impressive behavior is the fact that to generate HOMO and LUMO orbitals, which are required to obtain the HOMO−LUMO gap, we first generate the Fock matrix corresponding to the ML model, *f̂*[γ̂_*ML*_] and in a second step we diagonalize it to recover the canonical orbitals. As shown by the pioneering work of Harris,^60^ the diagonalization effectively leads to a better description of the 1-RDM compared to the predicted one.

We further examined the extrapolative performance of our models using methanol as test case, for which our training set contains structures sampling the three conformational minima. We investigated whether such training set is sufficient to accurately predict torsional barriers. We computed the energies along the CO rotational coordinate relative to the most stable conformation, see Figure 4c. As shown, both our ML models perform remarkably well, with errors on the order of 10^−3^ kcal/mol, even in the barrier region, where the extrapolative capability of the model solely determines the performance.

### 5.4 Stability of *Ab Initio* Dynamics and Accuracy of the Computed Atomic Forces

Stability of AIMD trajectories is directly related to the consistency between electronic energy and its gradient (atomic forces). If the energy gradients are not accurate, simulations in, e.g., the NVE ensemble become unstable beyond a characteristic instability time that is related to the error in the atomic forces. To assess force accuracy, we displaced biphenyl’s equilibrium geometry along its normal modes to sample 100 geometries corresponding to an effective temperature of *T* = 500, K, which is higher than both the training set and the subsequent AIMD simulation at *T* = 300, K. This higher temperature probes configurations well outside the training set, i.e., in the extrapolative regime. Forces were then computed using several ML models: the previously introduced B3LYP^ML^[γ_RBF_^*p*^], the δ-learning model B3LYP^ML^, and a force-corrected variant B3LYP^ML, corrected^[γ_RBF_^*p*^] (see Section 3.5). The results, summarized in Table S4 and Figure 5, show that the force correction reduces the RMSE by roughly an order of magnitude. Without correction, the error for biphenyl (trained on only 15k geometries) fluctuates randomly around zero with an RMSE of 10^−3^ Ha/Å, which is sufficient for geometry optimizations but not for stable, long AIMD runs. With force correction, the error decreases to an acceptable 10^−4^ Ha/Å. Interestingly, the δ-learning model yields an intermediate RMSE of 4 × 10^−4^ Ha/Å. Finally, Table S4 also reveals that doubling the training set size does not further improve the predictions.

**Figure 5:**
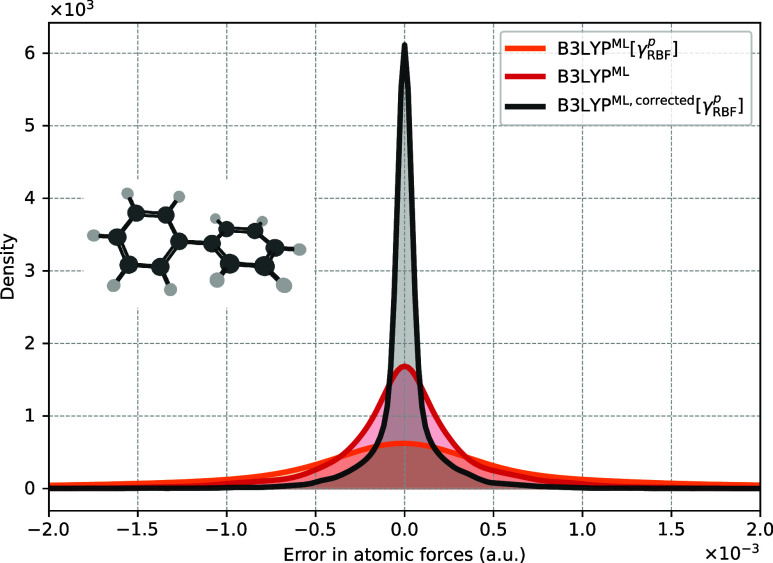
Error distributions of atomic forces predicted by different ML models for C_12_H_10_, compared with the B3LYP/6−31G* benchmark. RMSE and maximum absolute errors are summarized in Table S4.

The ultimate test involves evaluating if the models are able to yield stable AIMD simulations in the NVE ensemble. Additionally, and most importantly, we impose a very stringent test: the ability of the model to produce coherent AIMD trajectories compared to the reference QM method when the trajectories share the same initial conditions (same initial geometry and initial velocities). This is a very stringent test for any electronic structure method, as machine rounding errors introduce randomness in the computed energies and forces such that even if trajectories share the same identical initial conditions, they are bound to decohere after some time.

We ran a series of NVE trajectories for biphenyl all starting from the same initial conditions, see Figure 6. Different ML models were used, varying in kernel choice, training set size, and approach: B3LYP^ML^[γ^*p*^], B3LYP^ML^[γ^*p*^ + δγ^*p*^], and B3LYP^ML^ (LDA results are collected in Figure S2).

**Figure 6:**
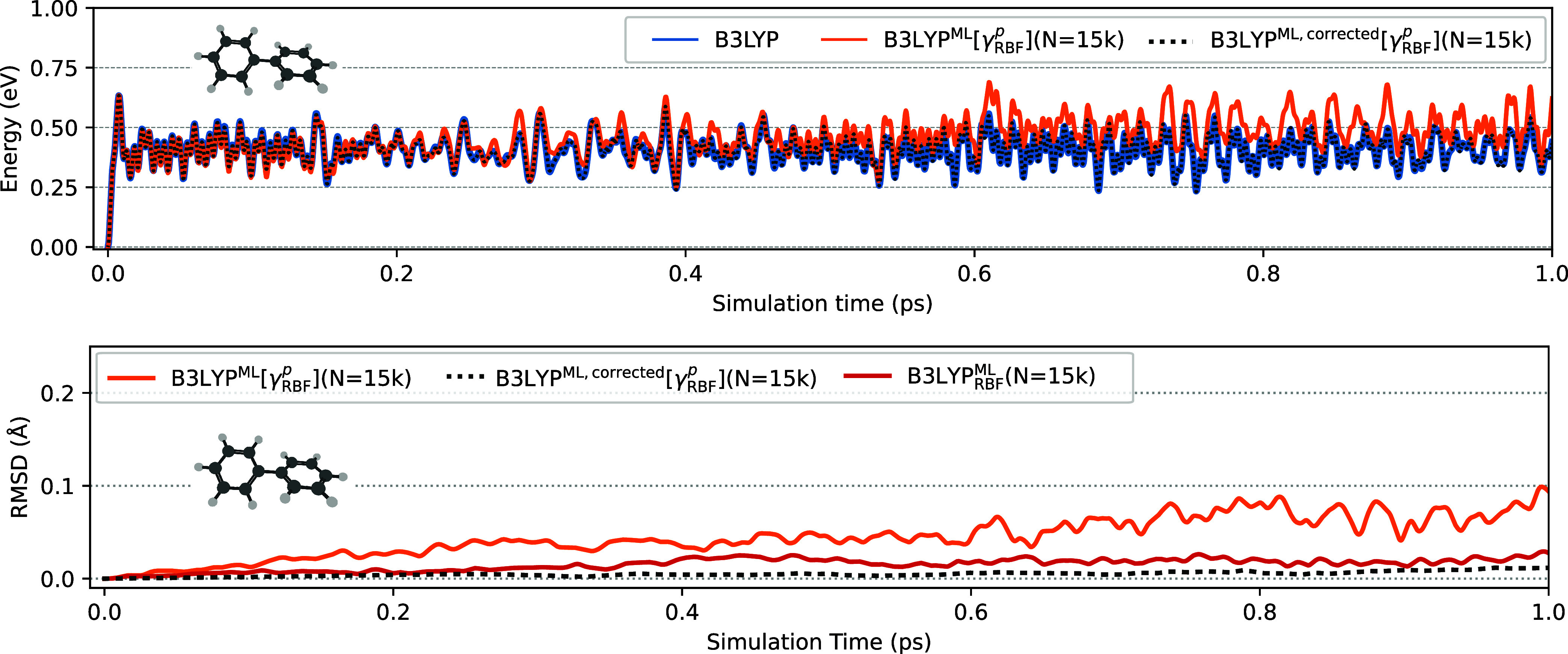
Comparison of the electronic energy (upper panel) and the trajectory decoherence (measured as the RMSD of the structures for the tractories compared to the reference trajectory) along AIMD trajectories in the NVE ensemble for the C_12_H_10_ molecule. All trajectories share the same initial conditions for initial geometry and velocities. The force-corrected trajectory (following 3.5) is also shown as the dotted black curve.

Figure 6 confirms the conclusions reached analyzing the accuracy of the atomic forces presented above. The force correction derived in Section 3.5 results in a stable trajectory closely following the B3LYP benchmark. In contrast, the method with uncorrected forces already decohered after about 0.2 ps and energy drifting after ∼ 600 fs. Increasing the training set to N = 30k reduces the drift significantly, though some deviations from the B3LYP benchmark still remain. We also found that imposing strict *N*-representability of the 1-RDM is crucial for maintaining stability during the AIMD (see Figure S3).

We additionally performed simulations using LDA and observed broadly similar behavior (Figure S2). In contrast to the RBF kernels, the decoherence between model and benchmark trajectories using linear kernels is quite severe (see Figure S4), making them less suitable for AIMD simulations.

We stress that even when employing the δ-learning method for energies and forces, the predicted 1-RDM is still available. As an example we show that the energies computed with the B3LYP^ML^[γ^*p*^ + δγ^*p*^] model along the B3LYP_LIN_^ML^ trajectory closely match the predictions made by the B3LYP_LIN_^ML^ model (see Figures S5 and S6).

### 5.5 Computational Cost

Figure 7 presents a comparison of the computational cost of the ML models against the target DFT method for biphenyl, using LDA and B3LYP functionals. The figure clearly shows that the models lead to substantial computational savings compared to the target electronic structure methods. This is so also for those models where the electronic energies and forces are computed from the ML-predicted 1-RDM, such as LDA^ML^[γ_RBF_^*p*^] and LDA^ML^[γ_LIN_^*p*^ + δγ_LIN_^*p*^] as well as the B3LYP versions which achieve at least a 5-fold speedup compared to DFT. The method employing δ-learning, LDA^ML^ and B3LYP^ML^ unsurprisingly yield the highest computational efficiency. Once again, this is so because the methods bypass the overhead associated with Fock builds and computation of forces and energies from the ML-predicted 1-RDM. Our modified protocol for computing atomic forces (see Section 3.5 and the black bars in Figure 7) only adds a small computational overhead to the overall workflow.

**Figure 7:**
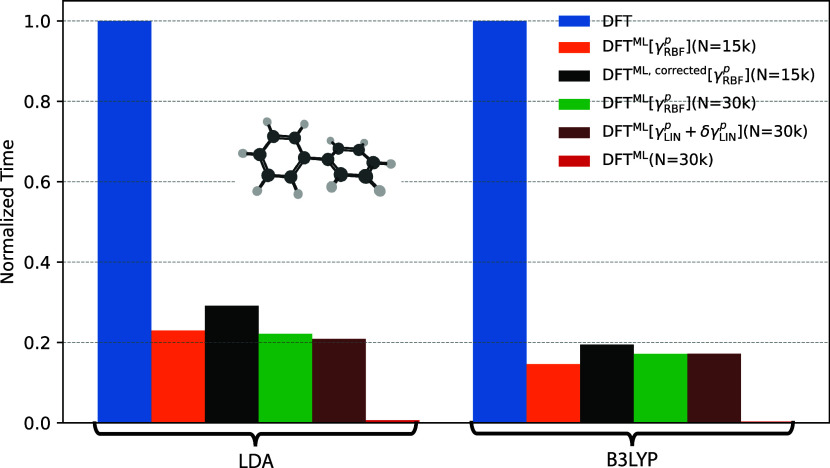
Computational cost for an AIMD simulations for the biphenyl molecule. Various ML models are compared against standard DFT dynamics performed at the LDA/6−31G* and B3LYP/6−31G* levels of theory. The costs are normalized to the DFT benchmark and averaged over 10 AIMD steps.

## 6 Conclusions

By employing kernel ridge regressions that learn the map connecting the 1-RDM with the external potential of an electronic system we developed surrogate models of commonly adopted electronic structure methods. The focus of this work is model optimization, training set convergence, assessment of the ability to drive ab initio dynamics and predict molecular properties.

Careful selection of kernels and KRR hyperparameters, when fully cross-validated, lead to models that require a limited, subcubic number of training structures and deliver 1-RDMs that are only about one SCF-threshold away from the target. Such accuracy is at least 3 orders of magnitude better than chemical accuracy. However, it must be reached if the models truly embody a surrogate electronic structure method.

Optimal models are found to be computationally cheap due to the simplicity of the underlying learning strategy (we use KRR) and due to the relatively small training set size. For example the models outperform the one previously published by our group^30^ while being computationally cheaper to train and at prediction.

We investigated the extrapolative behavior of our models. When we consider significantly distorted molecular geometries that lie outside the training set region, the model still accurately predicts energies, atomic forces and selected molecular properties (e.g., HOMO−LUMO gap).

Our models allow stable ab initio dynamics by merely plugging in the predicted 1-RDM into a DFT energy functional’s expression. The resulting atomic forces, properly corrected for the (even so slightly) nonvariational nature of the predicted 1-RDMs, give rise to trajectories that are stable and coherent with the benchmark when the benchmark is propagated from the same initial conditions.

Building on the promising performance of our models for isolated molecules, an obvious next step is to extend this framework to chemical reactions. This will require training data along the entire reaction coordinates, including reactants, products, intermediates and transition states. Incorporating such chemically diverse configurations represents an important avenue for future work and will broaden the applicability of this approach.

## Supplementary Material



## Notes

Software encoding our developments is freely available through Pypi and on GitHub.^38^ All training and testing data, as well as the associated codes and analysis scripts used in this study, are openly accessible on Zenodo at https://zenodo.org/records/17103131 (PDF)
