# Supplementary material for: Learning the One-Electron Reduced Density Matrix at SCF Convergence Thresholds
Source: J Chem Theory Comput. 2025 Dec 12;21(24):12652–63. doi: 10.1021/acs.jctc.5c01564 (PMC12746470; doi:10.1021/acs.jctc.5c01564)
Supplement: Supplementary file 1 [file ct5c01564_si_001.pdf]

Supplementary Information for: Learning the One-Electron Reduced Density Matrix at SCF Convergence Thresholds

Bhaskar Rana,[†,‖] Nicolas Viot,[†,‖] Jessica A. Martinez B.,[‡,†,‖] Xuecheng Shao,[¶] Pablo Ramos,[§,†] and Michele Pavanello[*,†,‡]

†*Department of Physics, Rutgers University, Newark, NJ 07102, United States*

‡*Department of Chemistry, Rutgers University, Newark, NJ 07102, United States*

¶*Key Laboratory of Material Simulation Methods & Software of Ministry of Education, College of Physics, Jilin University, Changchun 130012, PR China*

§*Division of Biology, Chemistry & Physics, Essex County College, Newark, NJ 07102, United States*

‖*These authors contributed equally to this work.*

E-mail: m.pavanello@rutgers.edu

Table S1: Energy RMSE associated to various supervised models of γ-learning for several small and medium sized molecules. The default hyperparameters are employed for all models (see scikit-learn's documentation for additional information).

**Model**	**Energy RMSE (kcal/mol/d.o.f.)**				
	CO_2	NH_3	CH_3OH	C_6H_6	$C_{10}H_8$
KRR	8.30E-04	1.29E-04	6.27E-04	7.36E-04	6.32E-04
Linear Regression	6.67E-04	1.43E-04	2.79E-03	1.75E-04	2.05E-04
OMP	6.67E-04	1.34E-04	6.11E-03	2.23E-03	2.80E-03
GPR	0.5672	0.1714	6.5518	0.2277	0.3717
Lasso	0.6438	0.1736	6.5758	0.2277	0.3717

Table S2: Energy RMSE associated to various supervised models of γ-learning for the H_2O molecule. As opposed to Table S1, the regularization parameters in the models are set to zero or the smallest value possible. These results show that certain models (such as Lasso) can be improved dramatically by tuning the involved hyperparameters. Additionally, the MLP and PLS models still show poor performance.

**Model**	**RMSE (Energy)** (kcal/mol/d.o.f.)
KRR	1.06E-04
Lasso	1.06E-04
OMP	1.13E-04
GPR	1.80E-04
Bayesian ARD	6.96E-04
GBR	0.0199
PLS	0.0502
Multilayer Perceptron (MLP)	0.0734

Table S3: Energy RMSE associated to various supervised models of δ-learning for several small and medium sized molecules. The default hyperparameters are employed for all models (see scikit-learn's documentation for additional information). KRR was used for the γ-learning step.

**Model**	**Energy RMSE (kcal/mol/d.o.f.)**				
	CO_2	NH_3	CH_3OH	C_6H_6	$C_{10}H_8$
Linear Regression	6.69E-04	1.41E-04	1.38E-04	1.82E-04	8.53E-05
GPR	6.69E-04	1.43E-04	1.06E-04	1.85E-04	9.22E-05
OMP	6.69E-04	1.36E-04	0.0020	9.94E-04	0.0011
KRR	0.4089	0.0686	0.0325	0.0022	0.0023
Lasso	0.6439	0.1736	6.5758	0.2277	N/A

Table S4: Root mean square error (RMSE) for dipole moment, energy, forces, and non-interacting kinetic energy (KE) predicted by the γ-learning step with several KRR kernels (LIN: linear, RBF, and POL: polynomial) with respect to target LDA results for all molecules. α is the kernel regularization parameter. All models use the same training set size. Color coding intensity is related to the magnitude of the RMSE per column.

Molecules	Model		Root Mean Square Error (RMSE)			
	KRR	α	Dipole (debye/dof)	Energy (kcal/mol/dof)	Forces (kcal/mol/Å/dof)	KE (kcal/mol/dof)
H_2O	LIN	0.1	0.5729	1.41E-04	0.5113	0.0979
	LIN	0.0	0.0518	1.06E-04	0.1147	0.0250
	RBF	0.0	0.0434	1.06E-04	0.1691	0.0265
	POL	0.0	0.0387	1.06E-04	0.1214	0.0433
NH_3	LIN	0.1	0.1917	1.29E-04	0.2898	0.0924
	LIN	0.0	0.0307	1.43E-04	0.0501	0.0092
	RBF	0.0	0.0093	1.43E-04	0.0285	0.0095
	POL	0.0	0.0132	1.43E-04	0.0289	0.0082
CO_2	LIN	0.1	0.2403	7.22E-04	0.8599	0.2148
	LIN	0.0	0.1157	6.69E-04	0.1262	0.0355
	RBF	0.0	0.0869	6.69E-04	0.1478	0.0372
	POL	0.0	0.0821	6.69E-04	0.1225	0.0385
CH_3OH	LIN	0.1	0.1338	4.22E-04	0.2327	0.1217
	LIN	0.0	0.0614	2.61E-04	0.0854	0.0408
	RBF	0.0	0.0449	1.29E-04	0.1582	0.0226
	POL	0.0	0.0500	1.15E-04	0.1061	0.0150
C_6H_6	LIN	0.1	0.1251	4.82E-04	0.1260	0.0373
	LIN	0.0	0.0462	1.71E-04	0.0846	0.0123
	RBF	0.0	0.0182	1.80E-04	0.0208	0.0059
	POL	0.0	0.0177	1.84E-04	0.0227	0.0059
1-C_3H_8O	LIN	0.1	0.3542	5.46E-03	0.1801	0.1807
	LIN	0.0	0.2303	8.49E-03	0.2163	0.1228
	RBF	0.0	0.0292	2.31E-05	0.0174	0.0187
	POL	0.0	0.0418	4.12E-04	0.0255	0.0257
2-C_3H_8O	LIN	0.1	0.3510	2.13E-03	0.1220	0.1379
	LIN	0.0	0.1931	1.10E-03	0.0793	0.0719
	RBF	0.0	0.0228	2.24E-04	0.0074	0.0097
	POL	0.0	0.0510	2.24E-04	0.0137	0.0219
$C_{10}H_8$	LIN	0.1	0.0784	3.94E-04	0.1265	0.0223
	LIN	0.0	0.0533	9.92E-05	0.0660	0.0109
	RBF	0.0	0.0237	7.61E-05	0.0322	0.0068
	POL	0.0	0.0281	7.61E-05	0.0337	0.0076
$C_{12}H_{10}$	LIN	0.1	0.0786	5.26E-04	0.0799	0.0173
	LIN	0.0	0.0469	3.07E-04	0.0426	0.0115
	RBF	0.0	0.0371	2.54E-04	0.0242	0.0072
	POL	0.0	0.0354	2.54E-04	0.0213	0.0064

Table S5: Same quantities as Table S4 and additionally the training set size used. The RBF model used a reduced training set size compared to Table S4. Results from two ML models are compared: $\mathrm{LDA}^{\mathrm{ML}}[\gamma^p_{\mathrm{RBF},\alpha=0.0}]$ (LIN) and $\mathrm{LDA}^{\mathrm{ML}}[\gamma^p_{\mathrm{LIN},\alpha=0.1} + \delta\gamma^p_{\mathrm{LIN}}]$ (RBF).

Molecules	Method			Root Mean Square Error (RMSE)			
	ML	N_{train}	N_{train} % Reduction	Dipole (debye/dof)	Energy (kcal/mol/dof)	Forces (kcal/mol/Å/dof)	KE (kcal/mol/dof)
H_2O	RBF	24	11%	0.0655	1.06E-04	0.1739	0.0288
	LIN	27	–	0.0544	1.06E-04	0.1767	0.0366
NH_3	RBF	195	10%	0.0098	1.43E-04	0.0390	0.0110
	LIN	216	–	0.0166	1.43E-04	0.0249	0.0057
CO_2	RBF	27	0%	0.0869	6.69E-04	0.1478	0.0372
	LIN	27	–	0.0443	7.13E-04	0.0931	0.0247
CH_3OH	RBF	5184	0%	0.0449	1.29E-04	0.1582	0.0226
	LIN	5184	–	0.0511	1.25E-04	0.0879	0.0159
C_6H_6	RBF	1000	93%	0.0888	1.73E-04	0.1077	0.0197
	LIN	13824	–	0.0226	1.80E-04	0.0359	0.0118
1-C_3H_8O	RBF	13824	67%	0.0631	2.63E-04	0.0206	0.0332
	LIN	41472	–	0.1606	9.01E-04	0.0783	0.0363
2-C_3H_8O	RBF	13824	67%	0.0532	2.24E-04	0.0155	0.0264
	LIN	41472	–	0.0774	2.58E-04	0.0342	0.0332
$C_{10}H_8$	RBF	4608	67%	0.0369	7.61E-05	0.0636	0.0099
	LIN	13824	–	0.0785	1.48E-04	0.0771	0.0153
$C_{12}H_{10}$	RBF	15000	50%	0.0435	2.62E-04	0.0325	0.0089
	LIN	30000	–	0.0328	2.53E-04	0.0213	0.0067

Table S6: RMSE and maximum absolute error of atomic forces (a.u.) for several ML models w.r.t. B3LYP/6-31G*. The test set comprises biphenyl geometries displaced along normal modes at an effective temperature of $T = 500\,\mathrm{K}$.

**Model**	N_{train}	Kernel type	RMSE (in a.u.)	Max Absolute Error (in a.u.)
$\mathrm{B3LYP}^{\mathrm{ML}}[\gamma^p]$	15k	RBF	1.71E-03	2.50E-02
$\mathrm{B3LYP}^{\mathrm{ML,\ corrected}}[\gamma^p]$	15k	RBF	2.11E-04	2.21E-03
$\mathrm{B3LYP}^{\mathrm{ML}}$	15k	RBF	4.12E-04	3.07E-03
$\mathrm{B3LYP}^{\mathrm{ML}}[\gamma^p]$	30k	RBF	7.69E-04	7.60E-03
$\mathrm{B3LYP}^{\mathrm{ML,\ corrected}}[\gamma^p]$	30k	RBF	9.12E-05	6.47E-04
$\mathrm{B3LYP}^{\mathrm{ML}}[\gamma^p]$	30k	LIN	3.80E-03	4.64E-02
$\mathrm{B3LYP}^{\mathrm{ML,\ corrected}}[\gamma^p]$	30k	LIN	5.52E-04	4.66E-03
$\mathrm{B3LYP}^{\mathrm{ML}}[\gamma^p + \delta\gamma^p]$	30k	LIN	1.02E-03	1.23E-02

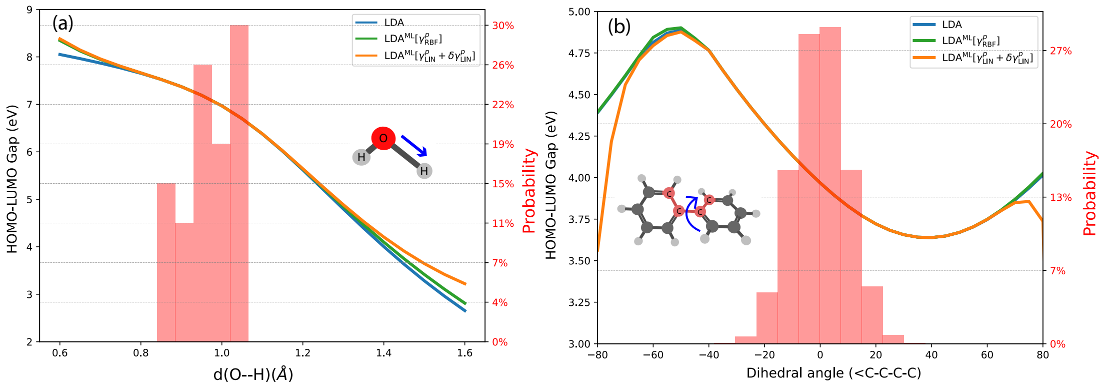

Figure S1: HOMO-LUMO gap for (a) a water molecule with geometries along the O-H bond stretch and (b) a biphenyl ($C_{12}H_{10}$) molecule with geometries following the dihedral angle (<C-C-C-C) twist, measured relative to the equilibrium structure (which corresponds to a dihedral angle of 38.49°). Results from two ML models are compared with the LDA calculations. The red histogram indicates the configuration space spanned by the training set structures.

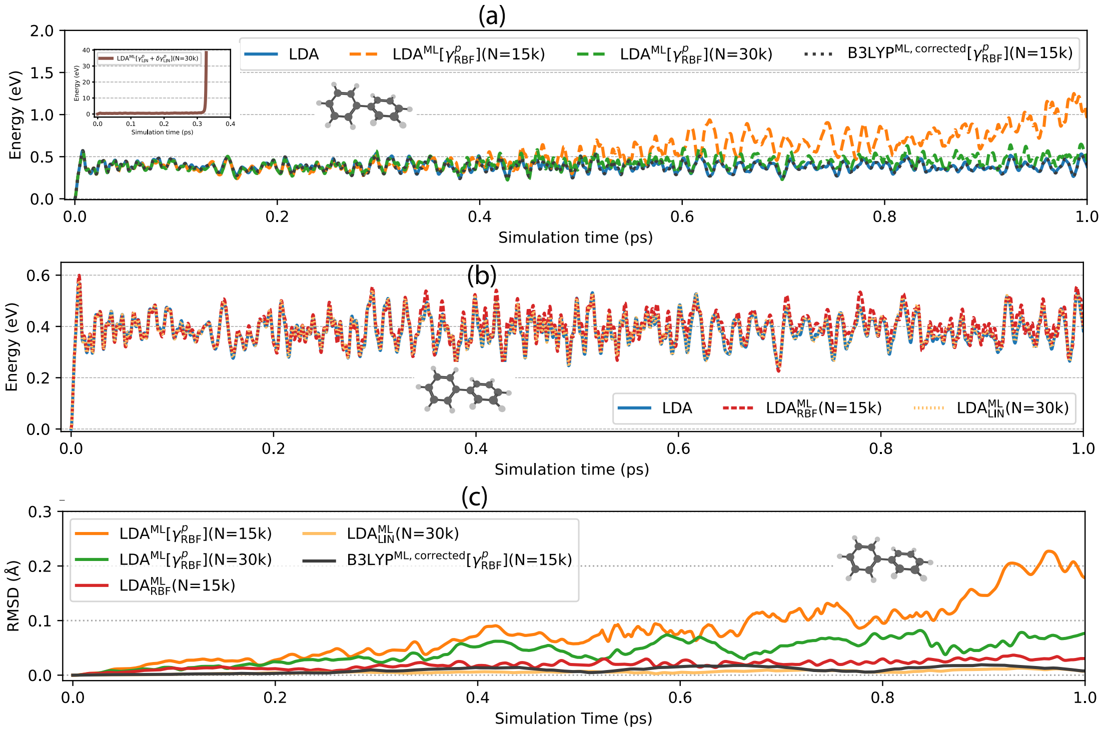

Figure S2: Comparison of energy conservation in a series of MD simulations for the $C_{12}H_{10}$ molecule, evaluating the performance of various ML models against the ab initio LDA results. (a) The performance of different surrogate ML models is analyzed, where energy and forces are computed from the ML-predicted γ using optimized RBF kernels with varying training set sizes. The linear kernel's performance, which diverges quickly, is shown in the inset for comparison. The effect of force correction on the stability of the AIMD simulation with our new protocol is also shown. (b) The $\text{LDA}^{\text{ML}}_{\text{RBF}}$ and $\text{LDA}^{\text{ML}}_{\text{LIN}}$ models are evaluated, where energy and forces are directly learned from γ through a secondary learning process. (c) Finally, the RMSD values of geometries obtained from various ML models are compared with those derived from LDA simulations.

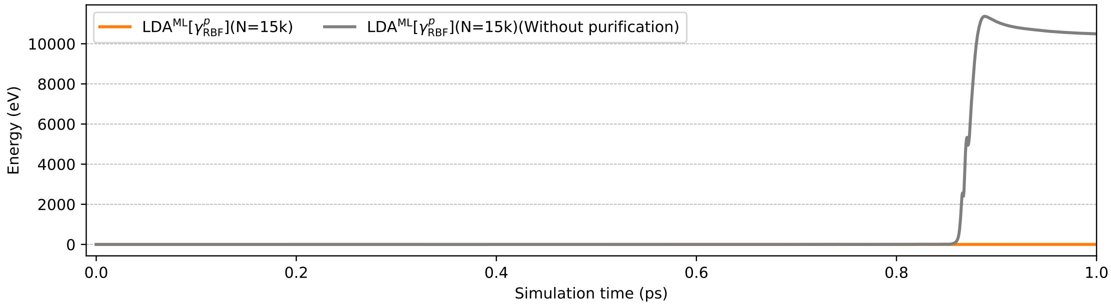

Figure S3: The importance of 1-RDM purification in our ML-based AIMD simulations for the $C_{12}H_{10}$ molecule is evident. The figure illustrates the results from an ML model using an RBF kernel for the γ-learning step. Without purification, the ML model produces unphysical geometries, and the relative energy spikes dramatically after approximately 850 fs. Similar instabilities have also been observed with other ML models.

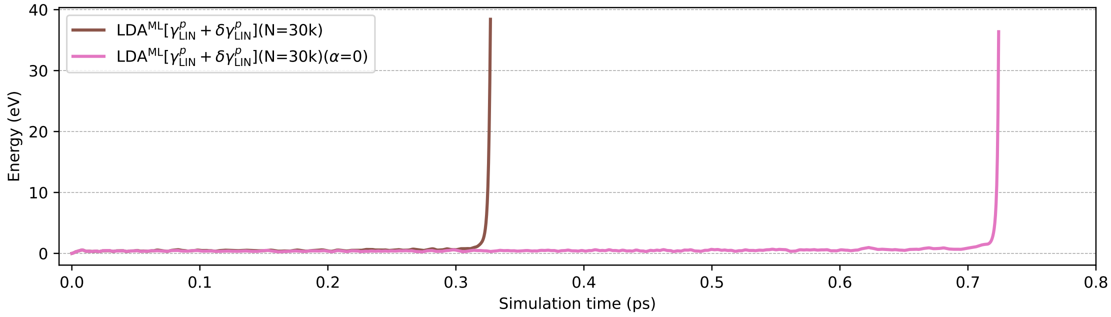

Figure S4: This figure compares energy conservation in MD simulations of the $C_{12}H_{10}$ molecule using ML models with linear kernels, both before and after optimizing the regularization parameter (α). In the original model ($\alpha = 0.1$), energy starts diverging after about 300 fs. Tuning the hyperparameters leads to $\alpha = 0.0$, which improves stability but only extends reliability up to around 700 fs.

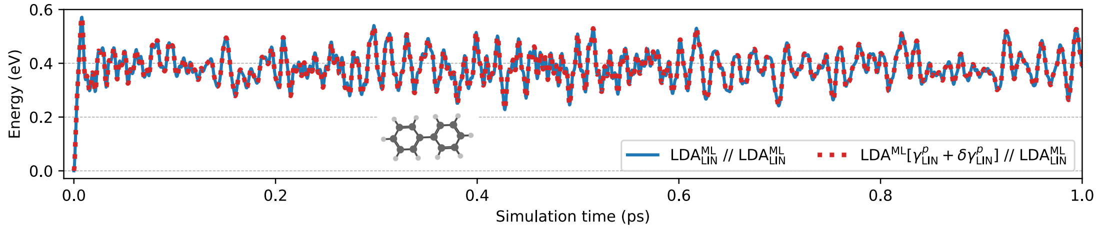

Figure S5: This figure compares the energies predicted by the LDA$^{\mathrm{LIN}}_{\mathrm{ML}}$ and LDA$^{\mathrm{ML}}[\hat{\gamma}_{\mathrm{LIN}} + \hat{\delta\gamma}_{\mathrm{LIN}}]$ models for the geometries encountered during the 1 ps long LDA$^{\mathrm{LIN}}_{\mathrm{ML}}$ MD trajectory.

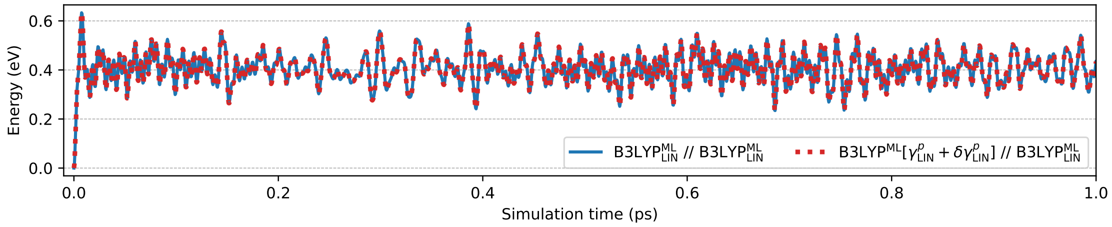

Figure S6: This figure compares the energies predicted by the $\text{B3LYP}^{\text{LIN}}_{\text{ML}}$ and $\text{B3LYP}^{\text{ML}}[\hat{\gamma}_{\text{LIN}} + \hat{\delta\gamma}_{\text{LIN}}]$ models for the geometries encountered during the 1 ps long $\text{B3LYP}^{\text{LIN}}_{\text{ML}}$ MD trajectory.

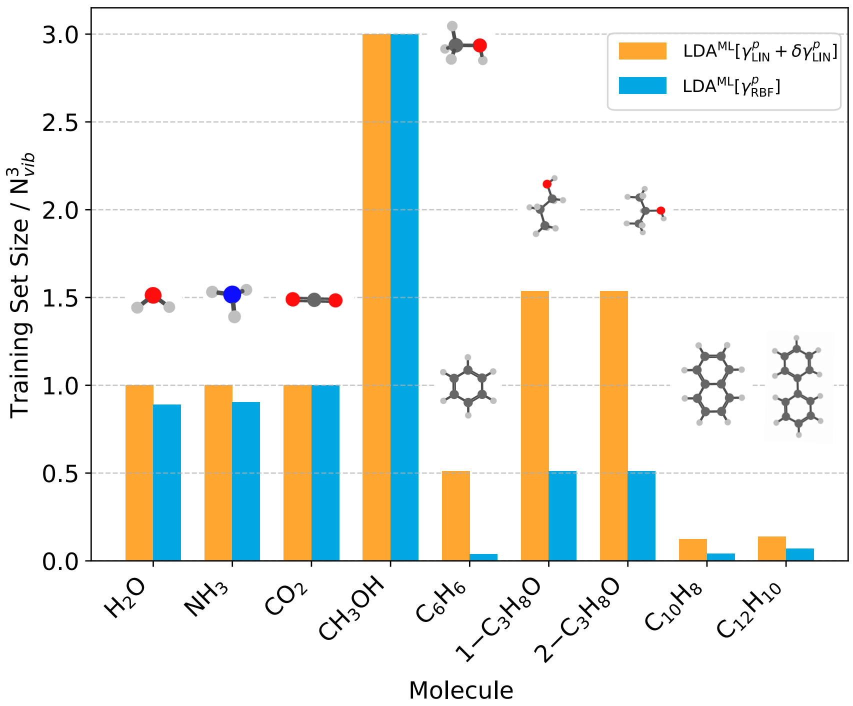

Figure S7: The normalized training dataset sizes for all molecules investigated in this work for LDA results. All values are normalized by N^3_{vib}, where N_{vib} is the number of vibrational modes. For raw values, see Table S5.

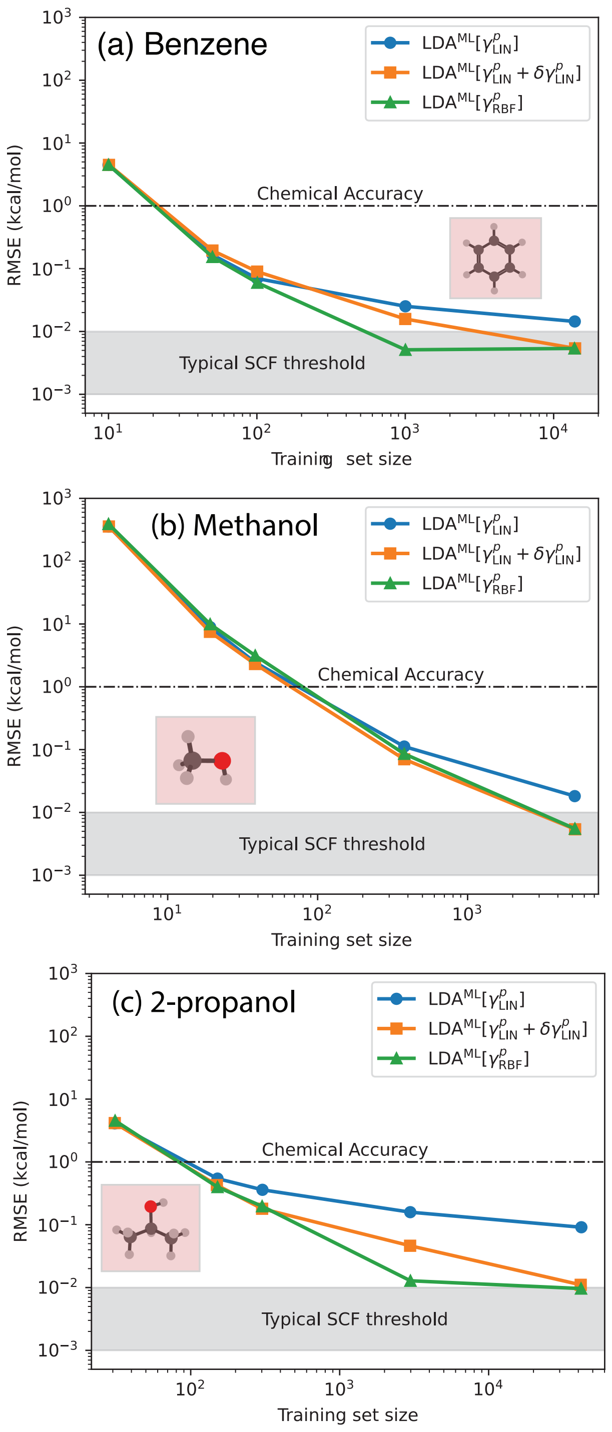

Figure S8: Energy RMSE for various ML models as a function of training set size for (a) benzene (C_6H_6), (b) methanol (CH_3OH), and (c) 2-propanol ($2\text{-}C_3H_8O$). The gray shaded area represents SCF-threshold accuracy, i.e., the accuracy we require from a surrogate model. This was done using LDA/6-31g* level of theory.
